# A systematic design method for robust synthetic biology to satisfy design specifications

**DOI:** 10.1186/1752-0509-3-66

**Published:** 2009-06-30

**Authors:** Bor-Sen Chen, Chih-Hung Wu

**Affiliations:** 1 Lab of Systems Biology, Department of Electrical Engineering, National Tsing Hua University, Hsinchu, 300, Taiwan

## Abstract

**Background:**

Synthetic biology is foreseen to have important applications in biotechnology and medicine, and is expected to contribute significantly to a better understanding of the functioning of complex biological systems. However, the development of synthetic gene networks is still difficult and most newly created gene networks are non-functioning due to intrinsic parameter uncertainties, external disturbances and functional variations of intra- and extra-cellular environments. The design method for a robust synthetic gene network that works properly in a host cell under these intrinsic parameter uncertainties and external disturbances is the most important topic in synthetic biology.

**Results:**

In this study, we propose a stochastic model that includes parameter fluctuations and external disturbances to mimic the dynamic behaviors of a synthetic gene network in the host cell. Then, based on this stochastic model, four design specifications are introduced to guarantee that a synthetic gene network can achieve its desired steady state behavior in spite of parameter fluctuations, external disturbances and functional variations in the host cell. We propose a systematic method to select a set of appropriate design parameters for a synthetic gene network that will satisfy these design specifications so that the intrinsic parameter fluctuations can be tolerated, the external disturbances can be efficiently filtered, and most importantly, the desired steady states can be achieved. Thus the synthetic gene network can work properly in a host cell under intrinsic parameter uncertainties, external disturbances and functional variations. Finally, a design procedure for the robust synthetic gene network is developed and a design example is given *in silico *to confirm the performance of the proposed method.

**Conclusion:**

Based on four design specifications, a systematic design procedure is developed for designers to engineer a robust synthetic biology network that can achieve its desired steady state behavior under parameter fluctuations, external disturbances and functional variations in the host cell. Therefore, the proposed systematic design method has good potential for the robust synthetic gene network design.

## Background

In short, synthetic biology is the engineering of biological systems to fulfill a particular purpose. It does so through transformative innovation that makes it possible to build living machines from off-the-shelf chemical ingredients, employing many of the same strategies that electrical engineers use to make computer chips [1]. The main goal of this nascent field of synthetic biology is to design and to construct biological systems with desired behaviors [2-5]. Drawing upon a set of the powerful techniques for the automated synthesis of DNA molecules and their assembly into genes and microbial genomes, synthetic biology envisions the redesign of natural biological systems for greater efficiency as well as the construction of functional "genetic circuits" and metabolic pathways for practical purposes [1,5]. Synthetic biology is foreseen to have important applications in biotechnology and medicine, and to revolutionize how we conceptualize and approach the engineering of biological systems [2].

At present, even the construction of networks of inter-regulating genes, i.e. the so-called genetic regulatory networks, has demonstrated the feasibility of synthetic biology [6]. The design of gene networks is still a difficult problem and the most newly designed gene networks cannot function properly. These design failures are mainly due to both intrinsic perturbations such as gene expression noises, splicing, mutation, evolution and extrinsic disturbances such as changing extra-cellular environments [2,7]. Therefore, how to design a robust synthetic gene network that can tolerate intrinsic parameter perturbations, attenuate extrinsic disturbances and also function properly in a host cell is an important topic of synthetic biology [2,7-9].

Previously, sensitivity analysis has been used for the analysis of the dynamic properties of gene networks either in the qualitative simulation of coarse-grained models or in the extensive numerical simulations of nonlinear differential equation models or stochastic dynamic models [10,11]. But for applications in synthetic biology, these approaches are not satisfactory since the local sensitivity analysis can provide only a partial description of all possible behaviors of the nonlinear gene network. In particular, it cannot guarantee that a synthetic network behaves as expected for all uncertain initial conditions, external disturbances and parameter variations in a given range. Moreover, obtaining all the convergences of states and parameter spaces by extensive numerical simulations quickly becomes computationally intractable as the size of the synthetic networks increases [7]. Recently, Kuepfer et al. have developed an approach based on semidefinite programming for partitioning parameter spaces of polynomial differential equation models into the so-called feasible and infeasible regions [12]. In this approach, 'feasible' simply refers to the existence of a desired steady state of the synthetic network. More recently, an approach using robustness analysis and tuning of synthetic networks was proposed by Batt and his colleagues to provide a means to assess the robustness of a synthetic gene network with respect to parameter variations [7]. This approach has the capability to search for parameter sets for which a given property is satisfied, using a publicly available tool called RoVerGeNe. Recently, several gene circuit design methods have been introduced to implement some circuits into or delete some circuits from an existing gene network to modify its structure so as to improve its robust stability and filtering ability [13,14]. However, the robust synthetic gene network design is a different topic. For that, it is necessary to design a completely man-made network with enough robust stability to tolerate parameter fluctuations and with enough noise filtering ability to resist the external disturbances so that it can work properly in the host cell. More recently, a robust synthetic biology design with molecular noises was developed based on stochastic game theory. However, the intrinsic parameter fluctuations of synthetic gene network have not been considered in the design procedure [15]. Further, some system design specifications may be given beforehand by users, so the designer must engineer an artificial gene network to meet these design specifications. Therefore, more efforts are needed to find effective design methods for robust synthetic gene networks.

Actually, many molecular-level processes of synthetic gene networks are deeply rooted in the statistical mechanical behaviors of so-called nanoscale biochemical systems, where the parameter fluctuations can be described by stochastic equations [16,17]. In this study, the design specifications of a robust synthetic gene network are given as follows. The variances of uncertain kinetic parameters and decay rates to be robustly tolerated are all specified beforehand by the designers according to the biological environment of the host cell. The steady states of a synthetic gene network are given according to the desired behaviors of some biotechnological purposes. The feasible ranges of kinetic parameters and decay rates to be designed are also specified beforehand according to the implementation ability of biotechnology. The effects of uncertain initial conditions, external disturbances and functional variations on the desired steady states of synthetic gene networks should be attenuated below a prescribed level. In other words, given the four design specifications, i.e., the tolerable variances of intrinsic stochastic parameter variations, the feasible ranges of kinetic parameters and decay rates, the desired filtering level of external disturbances, and the desired steady state, a robust synthetic gene network is designed to achieve the desired steady state that is guaranteed to satisfy these design specifications so that it can work properly in the host cell in spite of intrinsic uncertainties, extrinsic disturbances and variations of regulation functions.

The synthetic gene network with random parameter perturbations and external disturbances in the host cell can be described by a nonlinear stochastic system with state dependent noises and external disturbances. Then the design of robust synthetic gene networks essentially depends on how to specify some appropriate kinetic parameters and decay rates of gene networks to achieve the desired steady states despite uncertain parameter variations, external disturbances, and variations of regulation functions. In this study, the desired steady states are shifted to the origin of the system, and then the design problem of a robust synthetic gene network with a robust regulation to the desired steady state is equivalent to the robust stabilization and noise filtering design problems at the origin of the shifted gene network. Since the gene networks are inherently nonlinear stochastic systems, in order to avoid solving nonlinear Hamilton-Jacobin inequalities (HJIs) for robust stabilization and noise filtering design problems in the design procedure of nonlinear stochastic synthetic gene networks [18], global linearization techniques [19] are employed to simplify the design procedures of robust synthetic gene networks. The robust synthetic gene network design can be achieved by specifying suitable kinetic parameters and the decay rates of gene circuits by solving a set of linear matrix inequalities (LMIs), which are used to replace HJIs via the global linearization technique and can be efficiently solved using the LMI toolbox in Matlab [20]. Finally, an *in silico *design example of the robust synthetic gene network is given to illustrate the design procedure and to confirm the robust performance of the proposed design method under intrinsic parameter variations, external disturbances and variations of regulation functions.

## Methods

### Stochastic genetic network model and problem description

**Figure 1:**
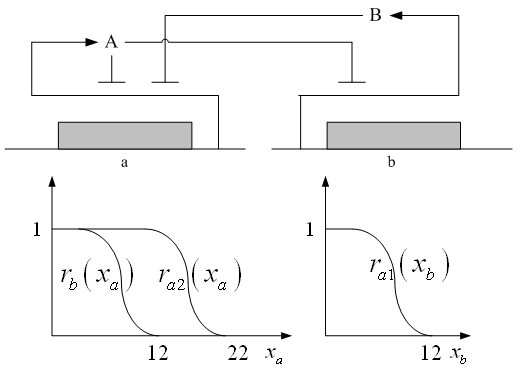
**A single two-genes network**. A simple two-genes cross-inhibition network and their regulation functions in (1) and (2).

Firstly, for the convenience of problem description, a simple design example [7] is provided to give an overview of the design problem for robust synthetic gene networks. A more general design problem of robust synthetic gene networks will be given in the sequel. First consider the cross-inhibition network shown in Figure 1. This network is synthesized with two genes, *a *and *b*, that code for two repressor proteins, *A *and *B*. More specifically, protein *B *represses the expression of gene *a*, whereas protein *A *represses the expression of gene *b*, and at higher concentration, the expression of its own gene. Protein degradations are not regulated. This synthetic system can be modeled by the following differential equations [7]

(1)

(2)

The state variables *x*_*a *_and *x*_*b *_denote the concentrations of proteins *A *and *B*. *κ's *and *γ's *are the kinetic parameters and decay rates, respectively, and *r's *are the regulation functions, which capture the regulator effect of an effector protein on gene expression and are smooth sigmoidal functions (e.g. Hill functions) [10,21].

The simple cross-inhibition network in (1) and (2) can be represented by the following stoichiometric matrix equation [22]:

(3)

However, the stoichiometric matrix *in vivo *will suffer from the intrinsic parameter perturbations due to gene expression noises, splicing, mutation, evolution, etc. as [16,21,23]

(4)

where Δ*κ*_*i *_and Δ*γ*_*i *_denote the amplitudes of fluctuations of the stochastic kinetic parameters and decay rates; and *n*_*i *_is a random white noise with zero mean and unit variance. Thus Δ*κ*_*i *_and Δ*γ*_*i *_denote the deterministic parts of parameter fluctuations and *n*_*i *_absorbs the stochastic property of intrinsic parameter fluctuations. The independent variables *n*_*a *_and *n*_*b *_indicate that there are two independent stochastic sources of random parameter fluctuations. The covariance of stochastic intrinsic parameter fluctuation Δ*κ*_*a*_*n*_*a *_is given as , where *δ*_*tτ *_denotes the delta function, i.e. *δ*_*tτ *_= 1 if *t *= *τ *and *δ*_*tτ *_= 0 if *t *≠ *τ*, i.e. Δ*κ*_*i *_denotes the standard deviation *σ*_*i *_of the stochastic parameter variation Δ*κ*_*i*_*n*_*i*_.

Suppose the synthetic gene network also suffers from environmental disturbances due to changing extra-cellular environments and interactions with the cellular context in its host cell. Then the stochastic gene network can be represented as

(5)

where *x*_2 _= [*x*_*a *_*x*_*b*_]^*T *^and *v*_2 _= [*v*_*a *_*v*_*b*_]^*T *^denote the state vector and the external disturbance of the synthetic gene network in the host cell, respectively. These intrinsic parameter fluctuations and external disturbances may cause the engineered gene network to be dysfunctional.

After employing the stochastic equation in (5) with intrinsic parameter fluctuations and external disturbances to mimic the realistic dynamic behaviors of the cross-inhibition network in the host cell, in order to work properly and efficiently, some design specifications for the synthetic gene network should be imposed as follows.

(i) The kinetic parameters and decay rates should be chosen from the following biologically feasible parametric ranges:

(6)

(ii) The intrinsic stochastic parameter fluctuations with the following standard deviations must be tolerated,

(7)

which are requested by designers to meet the in vivo conditions in the host cell.

(iii) The following desired steady states must be achieved to meet some bio-design purposes:

(8)

(iv) The external disturbances must be attenuated to remain below a prescribed attenuation level *ρ*, i.e. the effect of external disturbances on the regulation error around the desired steady state in (iii) should be less than *ρ*^2 ^from the mean energy point of view

(9)

for all possible bounded disturbances *v*_*a *_and *v*_*b*_. This is also a design specification for the noise filtering ability of the synthetic gene network, i.e. with a filtering ability of *ρ *to attenuate the external disturbances *v*_*a *_and *v*_*b *_[18,24]. In (9), we do not need to know the statistics of external disturbances *v*_*a *_and *v*_*b *_but are concerned only with the attenuation level (i.e. the ratio *ρ*) of external disturbances. If *v*_*a *_and *v*_*b *_are deterministic signals, the expectation *E *on *v*_*a *_and *v*_*b *_can be neglected.

Our design goal is to choose two kinetic parameters *κ*_*a *_and *κ*_*b *_and two decay rates *γ*_*a *_and *γ*_*b *_from the feasible parameter ranges in (6) so that the desired steady states *x*_*ad *_and *x*_*bd *_in (8) can be achieved under the above specified stochastic parameter variations and stochastic external disturbances, i.e. the allowable standard deviations of stochastic parameter fluctuations in (7) should be tolerated and the external disturbances should be attenuated below a prescribed attenuation level *ρ *in (9). If the above four design specifications (i)-(iv) can be imposed in the design procedure of the synthetic gene network, then the engineered synthetic gene network could work properly and efficiently in the host cell under intrinsic parameter fluctuations and external disturbances.

If a synthetic gene network consists of n genes, then the stochastic gene network of (5) in the host cell can be extended to the following n-gene network dynamics

(10)

where the state vector *x *= [*x*_1_...*x*_*n*_]^*T *^denotes the concentrations of proteins in the synthetic gene network. *N *denotes the corresponding stoichiometric matrix of the n-gene network. *M*_*i*_, *i *= 1,...,*m *denotes the fluctuation matrices due to independent random noise sources *n*_*i*_, *i *= 1,...,*m*, and the elements of *M*_*i *_denote the standard deviations of the corresponding parameter fluctuations. *v *= [*v*_1_...*v*_*n*_]^*T *^denotes the vector of external disturbances. The stochastic system in (10) is used to mimic the realistic dynamic behavior of a synthetic gene network of *n *genes in the host cell. This network, however, suffers from the intrinsic parameter fluctuations and external disturbances in the context of the host cell. Thus, a robust synthetic gene network should be designed with the ability not only to tolerate these parameter fluctuations and attenuate the external disturbances from the environments but also to achieve the desired steady state behaviors.

For convenience of analysis and design, the stochastic dynamic equation (10) of a more general stochastic gene network can be represented by the following Ito's stochastic differential equation [18,24,25]

(11)

where *W*_*i*_(*t*) is a standard Wiener process with *dW*_*i*_(*t*) = *n*_*i*_(*t*)*dt*.

The design specifications (6)-(9) can also be extended as follows for a more general synthetic gene network in (10):

(i) The kinetic parameters and the decay rates in stoichiometric matrix should be chosen from the following biologically feasible range

(12)

(ii) The stochastic kinetic parameters and decay rate fluctuations with prescribed standard deviations in *M*_*i *_in the following state-dependent noise terms

(13)

should be tolerated by the synthetic gene network.

(iii) The following desired steady state should be achieved

(14)

where *x*_*d *_is the desired steady state specified by the designer for some design purposes of the synthetic gene network.

(iv) The following prescribed disturbance filtering ability (i.e. the *H*_∞ _filtering) should be achieved [24]

(15)

for all bounded *v*(*t*), where *Q *≥ 0 is a symmetric weighting matrix and *ρ *is a prescribed attenuation level less than 1; i.e. the effect of external disturbance *v *on the regulation error *x *- *x*_*d *_should be less than the attenuation level *ρ *from the average energy perspective. In this situation, the synthetic gene network can efficiently attenuate the effect of external disturbances on the regulation of system state *x *to the desired steady state *x*_*d*_. The design specification of a prescribed attenuation level *ρ *may be a tradeoff between the filtering ability and the specification (i) in (12), i.e., a small *ρ *(i.e. a strict specification of attenuation level) may lead to a small feasible range of *N*, which may be outside the allowable range in (12). In (15), if the external disturbance is deterministic, then the expectation *E *can be neglected.

Based on the design specifications (12)-(15) of the stochastic gene network in (11), our design goal is to choose some kinetic parameters and decay rates in the stoichiometric matrix *N *from the biologically feasible parameter range [*N*_1_, *N*_2_] such that the desired steady state *x*_*d *_in (14) can be achieved, the stochastic parameter variations  can be robustly tolerated (stabilized), and the prescribed disturbance attenuation level *ρ *on *v *in (15) can be achieved. The design procedure for robust synthetic gene networks is described in the following section in detail.

## Results

### Design procedure for robust synthetic gene network

Based on the analyses in the above section, the design problem of robust synthetic gene networks becomes how to specify the kinetic parameters and decay rates in the stoichiometric matrix *N *in (11) such that the design specifications (12)-(15) must be satisfied to let the synthetic gene network work properly *in vivo *under intrinsic parameter fluctuations and external disturbances. In order to achieve the desired steady state *x*_*d*_, for the convenience of design, the origin of the nonlinear stochastic system in (11) should be shifted to *x*_*d*_. In such a situation, if the shifted nonlinear stochastic system is stabilized at the origin, then the desired steady state *x*_*d *_will be equivalently achieved. This will simplify the design procedure. Let us denote  = *x *- *x*_*d*_, then we get the following shifted stochastic system [26]

(16)

i.e. the origin  = 0 of stochastic system in (16) is at the desired steady state *x*_*d *_of the original stochastic system in (11).

For the stochastic system in (16), if we specify *N *∈ [*N*_1_, *N*_2_] such that the origin  = 0 can be robustly stabilized to tolerate the stochastic parameter fluctuation  and efficiently attenuate the external disturbance *v *to the following prescribed level (i.e. *H*_∞ _filtering ability)

(17)

then the design specifications (12)-(15) can be achieved for the stochastic gene network in (11) simultaneously under intrinsic parameter fluctuations and external disturbances in the host cell. If the initial condition is also considered [19,27], then the filtering ability in the inequality (17) should be modified as

(18)

for some positive function *V*().

According to the above analyses, we can design kinetic parameters and decay rates in *N *∈ [*N*_1_, *N*_2_] of the stochastic gene network in (16) to achieve both the robust stabilization to tolerate the stochastic parameter fluctuation and the filtering ability of external disturbance in (18). This is called the robust synthetic gene network design problem. Before further analysis of the robust stabilization and filtering design problem of stochastic synthetic gene networks, we first consider the robust stabilization to tolerate intrinsic stochastic parameter fluctuation in (16) in the case free from external disturbance (i.e. *v *≡ 0). From the theory of stochastic stability, the stochastic synthetic gene network in (16) with *v*(*t*) = 0 is assumed with asymptotic stability in probability if the expectation of the time derivative of Lyapunov (energy) function *V*() is negative [25,27], i.e.

(19)

where *V*() > 0 is the Lyapunov (energy) function of the synthetic gene network in (16). The inequality in (19) means that on average the energy function of the synthetic gene network decreases with time and will asymptotically converge to  = 0 or *x *→ *x*_*d *_in probability in the case of *v *= 0. In the case *v *≠ 0, only the *H*_∞ _disturbance attenuation level in (17) or (18) can be designed because the asymptotical stability in probability cannot be achieved due to the continuous interference of external disturbances, i.e.  → 0 or *x *→ *x*_*d *_cannot be achieved as *t *→ ∞ and the deviation from *x*_*d *_(i.e., ) due to external disturbances can only be attenuated to a level *ρ *by the design specification of noise filtering ability in (17) or (18).

From the stochastic network in (16), we obtain the following result:

#### Proposition 1

If some design kinetic parameters and decay rates in *N *∈ [*N*_1_, *N*_2_] are chosen such that the following Hamilton-Jacobi inequality (HJI) has a positive solution *V*() > 0

(20)

then (a) the stochastic gene network in (16) can achieve both the robust stabilization to tolerate intrinsic stochastic parameter perturbations and the prescribed attenuation level *ρ *on the external disturbances, i.e. the design specifications (i), (ii) and (iv) in (12), (13) and (15), respectively, are all satisfied; (b) if the stochastic gene network is free of external disturbances, i.e. *v*(*t*) = 0, then the shifted gene network in (16) will asymptotically converge to  = 0 or *x *→ *x*_*d *_in probability, or equivalently, the original stochastic gene network in (11) will asymptotically converge to the desired steady state *x*_*d *_in probability, i.e. the design specification (iii) in (14) is achieved.

**Proof: **See Appendix A

#### Remark 1

If the synthetic gene network is free of external disturbances and only the stochastic parameter fluctuations are to be robustly tolerated, the HJI in (20) is reduced to the following inequality

(21)

without the disturbance attenuation-related term . It is easier to find a positive solution *V*() > 0 to satisfy the HJI in (21) than in (20). Furthermore, the systematic gene network design which satisfies (21) could achieve asymptotical convergence in probability to the desired steady states in the disturbance free case.

In the above discussion, we only focus on the parameter perturbations which are allowed in the stoichiometric matrix of the nonlinear model and environment. Suppose the perturbations are also allowed in nonlinear functions governing the synthetic biological system, i.e. r-function in (1) and (2) also suffers from the stochastic perturbations *r*_*i*_(*t*) → *r*_*i*_(*t*) + Δ*r*_*i*_(*t*) such that *f*(*x*) and *g*_*i*_(*x*) of the nonlinear genetic system in (16) suffer from the stochastic perturbations *f*(*x*)→*f*(*x*) + Δ*f*(*x*) and *g*_*i*_(*x*)→*g*_*i*_(*x*)+Δ*g*_*i*_(*x*), respectively. In this situation, the nonlinear synthetic gene network suffers from the following parametric and functional perturbations

(22)

Suppose the functional perturbations are bounded by the following sectors,



or equivalently,

(23)

Then we can obtain the following result:

#### Proposition 2

Suppose the synthetic gene network suffers from the parametric variations and functional perturbations as (22) and (23). If some design kinetic parameters and decay rates in *N *∈ [*N*_1_, *N*_2_] are chosen such that the following HJI has a positive solution *V*() > 0

(24)

then there are two results: (a) the stochastic gene network in (22) can achieve *H*_∞ _robust stabilization to tolerate parametric variations and functional perturbation, and can reach the prescribed disturbance filtering ability *ρ *to attenuate the external disturbances; and (b) if the stochastic gene network in (22) and (23) is free of external disturbances, i.e. *v*(*t*) = 0, it will asymptotically converge to  = 0 or *x *→ *x*_*d *_in probability.

***Proof: ***See Appendix B

#### Remark 2

Comparing Proposition 1 and Proposition 2, it is seen that there are three extra terms in (24) due to the stochastic function perturbations. It is more difficult to find design parameters in *N *∈ [*N*_1_, *N*_2_] to solve *V*()>0 for HJI in (24) than to find parameters for HJI in (20) because the stochastic gene system in (22) has to tolerate not only the stochastic parameter variations but also the functional perturbations.

In general, it is very difficult to specify *N *∈ [*N*_1_, *N*_2_] to solve HJI in (20), (21) or (24) for *V*()>0 via the systematic method. At present, there is no good method to solve the nonlinear partial differential HJI analytically or numerically. In this situation, the global linearization technique is employed to transform the nonlinear stochastic gene network in (16) to an interpolation of a set of globally linearized gene networks to simplify the design procedure. By the global linearization method [19], if all the global linearizations are bound by a polytope consisting of *M *vertices as

(25)

where Co denotes the convex hull of polytope with *M *vertices defined in (25), then the state trajectories (*t*) of the shifted gene network in (16) will belong to the convex combination of the state trajectories of the following *M *linearized synthetic gene networks derived from the vertices of the polytope in (25) [19]

(26)

By the global linearization theory [19], if (25) holds, then every trajectory of the nonlinear synthetic gene network in (16) is a trajectory of a convex combination of *M *linearized synthetic gene networks in (26). Therefore, if we can prove that the convex combination of *M *linearized synthetic gene networks in (26) can tolerate the intrinsic parameter fluctuations and attenuate the external disturbances below a prescribed level, then the original nonlinear synthetic gene network in (16) will have the same robust stabilization and disturbance attenuation property. The convex combination of *M *linearized gene networks in (26) can be written as

(27)

where the interpolation function *α*_*j*_() satisfies 0 ≤ *α*_*j*_() ≤ 1 and , i.e. the trajectory of nonlinear synthetic gene network in (16) could be represented by the interpolated synthetic gene network in (27), which is the convex combination of *M *linearized gene networks in (26).

Based on the global linearization theory [19], if we can prove that the synthetic gene network consisting of the convex combination of *M *linearized gene networks in (26) can be robustly stabilized under intrinsic parameter noises and achieves a prescribed disturbance attenuation level in (17) or (18), so will be the nonlinear gene network in (16). Therefore, we obtain the following result.

#### Proposition 3

Assume that some design kinetic parameters and decay rates in *N *∈ [*N*_1_, *N*_2_] are chosen such that the following *M *inequalities have a common symmetric positive definite solution *P *> 0

(28)

then there are two results: (a) the stochastic gene network with parametric variations and external disturbances in (16) will be robustly stable to tolerate intrinsic stochastic parameter perturbation and also achieves a prescribed attenuation level *ρ *on the external disturbance, i.e. the design specifications (i), (ii) and (iv) in (12), (13) and (15) are all satisfied; and (b) if the gene network is free of external disturbance, i.e. *v*(*t*) = 0, then the gene network in (16) will asymptotically converge to  = 0 in probability, or equivalently, the original synthetic gene network in (11) will asymptotically converge to the desired steady state *x*_*d *_in probability, i.e. the design specification (iii) in (14) is achieved.

***Proof: ***See Appendix C.

Similarly, for the stochastic gene network in (22) with parameter variations, functional perturbations and noises, based on global linearization method, we obtain the following result for Proposition 2

#### Proposition 4

Assume some design kinetic parameters and decay rates in *N *∈ [*N*_1_, *N*_2_] are chosen such that the following *M *inequalities have a common symmetric positive definite solution *P *> 0

(29)

then there are two results: (a) the synthetic gene network with parameter variations, functional perturbations and external disturbances in (22) will be robustly stable to tolerate intrinsic parameter variation and functional perturbations, and achieve a prescribed attenuation level *ρ *on the external disturbances; and (b) if the synthetic gene network is free of external disturbances, then the synthetic gene network in (22) will asymptotically converge to  = 0 in probability, or *x *→ *x*_*d *_asymptotically in probability.

***Proof: ***Similar to Proposition 3

#### Remark 3

(i) By Schur complement [19], the inequalities in (28) could be transformed to the following LMIs

(30)

The robust synthetic gene network design problem by specifying *N *∈ [*N*_1_, *N*_2_] to solve a positive function *V*() > 0 for HJI in (20) with a prescribed disturbance attenuation level *ρ *is transformed into the problem of specifying *N *∈ [*N*_1_, *N*_2_] to solve a common positive symmetric definite matrix *P *> 0 for a set of inequalities in (28), or equivalently for a set of LMIs in (30). The LMIs in (30) can be efficiently solved by the so-called interior-point method [19]. It has been proven that the computational complexity for solving LMIs in (30) via the interior point method for the n-gene network in (10) is about the order *O*(*m*^2.75 ^*M*^1.5^) of arithmetic operations, where  and *M *is the number of linearized systems [19]. The LMIs in (30) could be efficiently solved by the LMI toolbox in Matlab [20].

Similarly, by Schur complement [19], the inequalities in (29) are equivalent to specifying *N *∈ [*N*_1_, *N*_2_] to solve *P *> 0 for the following LMIs

(31)

(ii) If the synthetic gene network is free of external disturbances and the robust stabilization only needs to tolerate the stochastic parameter fluctuation, then the inequalities in (28) will be reduced to the following LMIs

(32)

without the term  in (28). In this situation, it is easier to specify the kinetic parameters and decay rates in *N *to satisfy the above LMIs. Furthermore, the asymptotic convergence to the desired steady states *x*_*d *_in probability can also be achieved.

(ii) In addition to the global linearization method in this study, a piecewise-affine model for nonlinear gene regulatory network has also been introduced to consider geometric constraints of genetic regulatory network [28].

(iv) Using global linearization, every trajectory of a nonlinear system in (16) is also a trajectory of the convex combinatory system in (26). However, there are many trajectories of the convex combinatory system that are not trajectories of the nonlinear system [19]. Therefore, the conditions of Proposition 3 are more constraining than the ones of Proposition 1. Hence, the solution of Proposition 3 is more conservative than the one of Proposition 1. Similarly, the solution of Proposition 4 is more conservative than the one of Proposition 2 because the conditions of Proposition 4 are more constraining than the ones of Proposition 2.

Based on the above analyses, the design problem of robust synthetic gene network becomes how to select an adequate *N *from the allowable range [*N*_1_, *N*_2_] to satisfy the LMIs in (30) to meet the design specifications (i)-(iv) in (12)-(15). In order to simplify the selection process of *N*, we define

(33)

where *N*_0 _denotes the nominal value and  denotes the allowable range from the nominal value. Let

(34)

i.e. we could select the nominal *N*_0 _for *N *at first and then add a fine tuning  around the nominal *N*_0 _to meet LMIs in (30) or we could select fine tuning  to meet the following LMIs to simplify the design procedure.

(35)

Then developing the robust synthetic network requires finding a fine tuning Δ*N *from the allowable range [-, ] to meet a positive matrix *P *> 0 solution of LMIs in (35), which can be achieved via the help of the LMI toolbox in Matlab. The detailed search process for fine tuning Δ*N *is given in the design example in the sequel.

From the analyses above, a design procedure for a robust synthetic gene network is proposed as follows:

(1) Provide the design specification of robust synthetic gene network in (12)-(15).

(2) Shift the desired steady state *x*_*d *_to the origin, as in (16).

(3) Perform the global linearization as in (25) to obtain *F*_*i *_and *G*_*ij*_.

(4) Find the nominal  and solve LMIs for fine tuning Δ*N *from the allowable range (-, ).

(5) Find the design kinetic parameters and decay rates of the synthetic gene network as *N *= *N*_0 _+ Δ*N*.

### An *in silico *design example

**Figure 2:**
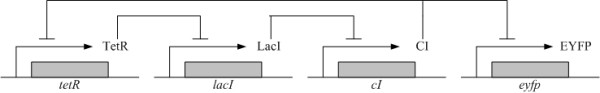
**Synthetic transcriptional cascade loop**. Synthetic transcriptional cascade loop *in silico *design example. TetR represses *lacI*, LacI represses *cI*, and CI represses *eyfp *and *tetR*. The fluorescent protein EYFP is the output.

After introducing the design procedure of a robust synthetic gene network with the ability to tolerate the intrinsic parameter fluctuation and to attenuate the external disturbance in the above section, an *in silico *design example is introduced here to illustrate the design procedure for a robust synthetic gene network and to confirm the robust stabilization and disturbance attenuation performance of the proposed robust design method. We want to synthesize a cascade loop of transcriptional inhibitions built in *E. coli*. [29]. The synthetic gene network is represented in Figure 2. It consists of four genes: *tetR*, *lacI*, *cI*, and *eyfp *that code for three repressor proteins, TetR, LacI and CI, and the fluorescent protein EYFP, respectively [7]. The fluorescence of the system, due to the protein EYFP, is the measured output. The protein CI inhibits gene *eyfp *and gene *tetR*. The protein TetR inhibits gene *lacI*. The protein LacI inhibits gene *cI*. The regulatory dynamic equations of the synthetic transcriptional cascade in Figure 2 are given as follows [7].

(36)

where *κ*_*tetR*,0_, *κ*_*lacI*,0_, *κ*_*cI*,0_, and *κ*_*eyfb*,0 _are the nominal generating ratios of the corresponding proteins, which are assumed to be 150, 587, 210, and 3487, respectively, but with stochastic parameter fluctuations. In addition, *κ*_*tetR*_, *κ*_*lacI*_, *κ*_*cI*_, and *κ*_*eyfb *_and *γ*_*tetR*_, *γ*_*lacI*_, *γ*_*cI*_, and *γ*_*eyfb *_are, respectively, the kinetic parameters and decay rates of the corresponding proteins, which also suffer from parameter fluctuations in the host cell (i.e. *E. coli*.) and are to be specified to meet the four design specifications (12)-(15). Furthermore, r_*tetR*_(*x*), *r*_*lacI*_(*x*), *r*_*cI*_(*x*), and *r*_*eyfp*_(*x*) are the Hill functions for repressors. The Hill function is a decreasing S-shaped curve, which can be described in the form  with *β *= 1, *n *= 2, *K*_*i *_= 1000, *i *= *tetR*, *lacI*, *cI*, *eyfp *[10,21].

According to the stochastic gene network in (11), the stochastic gene network with four random parameter fluctuation sources in (36) can be represented by

(37)

Our robust synthetic gene network requires designing these parameters *κ*_*i *_and *γ*_*i *_within *N *∈ [*N*_1_, *N*_2_] to meet the four specifications, i.e., we want to design four kinetic parameters *κ*_*tetR*_, *κ*_*lacI*_, *κ*_*cI*_, and *κ*_*eyfp *_and four decay rates *γ*_*tetR*_, *γ*_*lacI*_, *γ*_*cI*_, and *γ*_*eyfp *_to satisfy the following four design specifications.

(i) Suppose the biological allowable ranges of kinetic parameters and decay rates to be designed are given by [7]

(38)

where the allowable ranges of kinetic parameters and decay rates depend on the possibility of implementation and the biological property such as the desired steady state *x*_*d*_.

(ii) The standard deviations of parameter fluctuations to be tolerated are given as

(39)

(iii) The desired steady state *x*_*d *_is given by [7]

(40)

(iv) The prescribed attenuation level of external disturbance is specified by *ρ *= 0.3.

Based on the design procedure, we first shift the desired steady state *x*_*d *_of the synthetic gene system to the origin, then perform the global linearization to obtain *F*_*i *_and *G*_*ij *_for *i *= 1,...,3, *j *= 1,...,4 (see Appendix D), and finally solve LMIs for fine tuning parameters. The allowable range [*N*_1_, *N*_2_] has been obtained by the parameter-range specification in (i). In order to simplify the selection process of *N*, we get  and  as



By solving LMIs for fine tuning Δ*N *from the allowable range (-, ), we find a positive definite matrix *P *of LMIs in (35) if the allowable range is distributed over [-Δ*N*, Δ*N*] with



i.e., if the design kinetic parameters *κ*_*i *_and decay rates *γ*_*i *_of the synthetic gene network are specified within the following ranges:

(41)

then the four design specifications (i)-(iv) are satisfied.

In order to confirm the performance of the proposed robust synthetic gene network, we design the synthetic gene network with the set of kinetic parameters *κ*_*i *_and decay rates *γ*_*i *_in the ranges given in (41) to see if they can achieve the desired steady state in spite of initial conditions, parameter fluctuations and extrinsic disturbances. Let us choose the following design parameters from the ranges given in (41).

(42)

**Figure 3:**
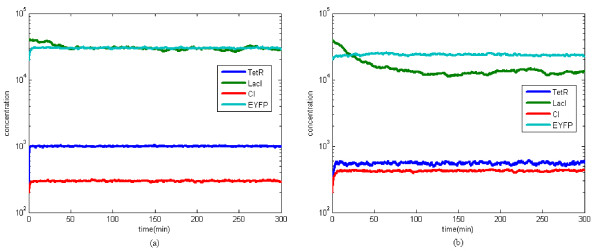
**Simulation**. In order to confirm the stability robustness and filtering ability of the synthetic gene network *in silico *example, we simulate the synthetic gene network with the initial value [200, 40000, 200, 20000] and the desired steady state [1000, 30000, 300, 30000]. (a) with the design parameters (*κ*_*tetR*_, *κ*_*lacI*_, *κ*_*cI*_, *κ*_*eyfp*_) = (2000, 2000, 2000, 15000) and (*γ*_*tetR*_, *γ*_*lacI*_, *γ*_*cI*_, *γ*_*eyfp*_) = (1.98, 0.05, 0.7, 0.57) in the specified parameter range given in (41), it is seen that the synthetic gene network has robust stability and noise filtering ability to achieve the desired steady state in spite of parameter fluctuations and disturbances in the host cell. (b) If the design parameters are outside the specified range with (*κ*_*tetR*_, *κ*_*lacI*_, *κ*_*cI*_, *κ*_*eyfp*_) = (150, 100, 500, 1500) and and (*γ*_*tetR*_, *γ*_*lacI*_, *γ*_*cI*_, *γ*_*eyfp*_) = (0.5, 0.05, 0.5, 0.2), the expression of the synthetic gene network is with more fluctuation and cannot achieve the desired steady state under parameter fluctuations and environmental disturbances.



The desired steady states can be achieved under intrinsic parameter fluctuations and extrinsic disturbances by the proposed robust synthetic gene network design method. From the simulation in Figure 3a with *v*(*t*) = [10*n*_1_, 1000*n*_2_, 10*n*_3_, 1000*n*_4_], where *n*_*i*_, *i *= 1,...,4 are independent Gaussian white noises with unit variance. The disturbance attenuation level of external disturbance, which is prescribed by *ρ *= 0.3, is estimated as

Clearly, the prescribed disturbance attenuation (filtering ability) is achieved by the proposed method.

In contrast to the above design case, we also design the synthetic gene network with parameters outside the ranges in (41), for example, with kinetic parameters *κ*_*i *_= (150, 100, 500, 1500) and decay rates *γ*_*i *_= (0.5, 0.05, 0.5, 0.2), which are outside the specified regions in (41). The simulation is shown in Figure 3b. Obviously, the time response of the synthetic network suffers more external disturbances and cannot achieve the desired steady states. In this design case, the disturbance attenuation level of external disturbance is estimated as:



Clearly, the design specification of filtering ability is violated significantly.

From the simulation results, it can be seen that the designed synthetic gene network using the proposed method has robust stability to tolerate intrinsic parameter fluctuations and enough filtering ability to attenuate the external disturbances, thereby achieving the desired steady states. If the designed gene network has enough robust stability and filtering ability, then it could work properly under intrinsic fluctuations and extrinsic molecular noises on the host cell. Furthermore, the design ranges of kinetic parameters and decay rates can be easily solved by fine tuning Δ*N *in the design procedure using the LMI Toolbox in Matlab.

## Discussion

In this design example, the kinetic parameters *κ*_*i *_and decay rates *γ*_*i *_can be designed within the ranges in (41) to satisfy the four design specifications. Recently, the gene circuit design can be implemented using a recombination technology [30,31] to insert or delete TF binding sites in the promoter region of a regulated gene to increase or decrease the value of the kinetic parameter *κ*_*i *_(i.e. different levels of affinity) of the regulated gene. By inserting strong or weak binding sites, we can get a large or small kinetic parameter *κ*_*i*_. For example, the binding site of *κ*_*i *_= 1 will be 10 times larger than that of *κ*_*i *_= 0.1 at the promoter region of target gene *i*. As for the implementation of decay rate *γ*_*i*_, it has been shown that it can be achieved by shortening the 3' polyadenylate tail (referred to as deadenylation), which primarily triggers decapping, leading to 5' to 3' exonucleolysis. Alternatively, the removal of 3' polyadenylate tail can facilitate the decay rate *γ*_*i *_[32,33]. Therefore, by shortening or elongating the gene's 3' polyadenylate tail we can increase or decrease the decay rate *γ*_*i *_of gene *i*. Recently, the directed evolution methods are also used to change the elasticity (kinetic property of *κ*_*i*_) and will be useful techniques for biochemical circuit design [34-36]. The advances of implementation techniques of kinetic parameter *κ*_*i *_and decay rate *γ*_*i *_have made an engineering of synthetic gene network possible in the near future [32].

The synthetic gene network *in silico *example is a negative circuit made of the sequence inhibitions of three repressor genes and an output to reveal the gene expression state *in vivo*. i.e., *eyfp *and *tetR *expressions are controlled by CI protein, *cI *expression is controlled by LacI protein, which is under the control of TetR protein, and enhanced yellow fluorescent protein (EYFP) is the output. In general, negative feedback can reduce noise and introduce stability [37], but it can also generate oscillations if a long time delay is involved [38,39]. Therefore, proper kinetic parameters and decay rates in synthetic gene network are important to satisfy the four design specifications proposed for robust stabilization and filtering ability. Specification (iii) delivers the desired steady state *x*_*d *_given in (40) to validate the robust regulation of synthetic gene network. Nevertheless, regulation of gene expression is an evolutionary response to the challenge of surviving in a changing environment [40], the generating ratios *κ*_*i*,0_, the kinetic parameters *κ*_*i*_, and the decay ratios *γ*_*i *_of the corresponding proteins in a synthetic gene network may tolerate parameter fluctuations in the host cell, but it is difficult to maintain the values of these parameters invariant *in vivo*. Therefore, to remedy these uncertainties in the host cell, specifications (ii) and (iv) provide the standard deviations of parameter fluctuations in (39) and the prescribed attenuation level *ρ *to contribute to the guaranty of stability robustness and filtering ability of the synthetic gene network. By using the above design specifications and the robust design method, biologists may select suitable ranges of kinetic parameters and decay rates to design a robust synthetic gene network to meet these design specifications. Therefore, the proposed robust design method has several applications to robust synthetic gene network design in the near future.

Due to intrinsic perturbations such as gene expression noises, mutation, evolution and extrinsic disturbances such as changing environments and interactions with the cellular context in a host cell, the synthetic gene networks engineered so far in bacteria to behave in a particular way seem decay rapidly after a short period of activity [39,41]. Therefore, the development of a robust design scheme is an important topic for synthetic gene network to work properly and robustly in spite of intrinsic parameter fluctuations, external molecular noises and functional variations in the host cell. If successful, the behavior of the synthetic gene network can be maintained in spite of environmental factors. In [42-49], robust gene circuit designs have been proposed to attenuate the parameter variations or noises. However, in this study, the stochastic parameter fluctuations are modeled as state-dependent Wiener noises due to several independent random sources in the host cell and environmental disturbances are to be attenuated below a prescribed level so that a synthetic gene network can be robust in the host cell. Further, we propose four design specifications for engineering synthetic gene network to guarantee this robust design purpose. Then, based on global linearization and LMI techniques, a simple design procedure is developed to achieve the robust design purpose of synthetic gene network using the LMI Toolbox in Matlab. From the design example *in silico*, the four design specifications can be guaranteed for the robust synthetic gene network via the computation simulation.

There are, however, many other behaviors of interest in synthetic gene network designs to which the proposed method with desired steady state behavior can not be applied. Indeed, the other behaviors like oscillations or transient behaviors are more complex than the steady state behaviors. How to engineer a synthetic gene network with desired oscillations or transient behaviors is a tracking design problem. In this tracking design case, the desired behavior should be generated by a reference dynamic model , where *x*_*d *_denotes the desired state vector and *r *denotes the reference input. Then we should design the parameters of the synthetic gene network in (11) so that *x *can track the reference state vector *x*_*d *_and the prescribed disturbance filtering ability *ρ *in (15) can be achieved. This is a desired behavior tracking design problem. More effort is needed and this will be our further research in future. The proposed design method only focuses on the regulation problem of the desired steady state behaviors, which is a limitation of the approach for the general problem of synthetic biological network design.

## Conclusion

We have presented a stochastic model to analyze the dynamic properties of genetic regulatory networks with parameter uncertainties, external disturbances and functional variations in the host cell. Then four design specifications are introduced to guarantee that synthetic gene networks could work with the desired steady state behaviors under intrinsic parameter fluctuations, external disturbances and functional variations in the host cell. Finally, a synthetic design method is proposed for robust synthetic gene networks to meet these design specifications and function properly in spite of intrinsic parameter fluctuations and extrinsic disturbances. To avoid directly solving nonlinear stochastic stabilization and the filtering design problem of robust synthetic gene networks, a global linearization technique is employed to transform a nonlinear stochastic gene network to the set of linearized gene networks to simplify the design procedure so that the robust synthetic gene network design problem could be solved efficiently by linear matrix inequalities (LMIs) technique using the LMI toolbox in Matlab. The proposed design procedure can guarantee that the synthetic gene network meets the four design specifications so that the engineered gene network has sufficient robust stability and filtering ability to achieve the desired steady state in spite of intrinsic uncertainties, extrinsic disturbances and functional variations in the host cell. Therefore, the proposed systematic design method has significant potential for application to robust synthetic gene network designs in the future.

## Authors' contributions

BSC gave the topic, derived some design methods and was involved in drafting the manuscript, and CHW performs the design, simulations and revising.

## Appendix A: proof of proposition 1

Let us choose a Lyapunov function *V*() > 0 with *V*(0) = 0

(A1)

By the Ito's formula [25] and , we get

(A2)

Substituting (A2) into (A1), we get

(A3)

By the inequality (20), we have

(A4)

If *V*((0)) = 0, then (A4) will be reduced to (17).

If the synthetic gene network is free of the external disturbances, i.e *v *= 0, then we have

(A5)

For some positive constant *V*((0)), it means  → 0 in probability as *t *→ ∞

## Appendix B: proof of proposition 2

Let us choose a Lyapunov function *V*()>0 for the stochastic gene network in (22). By the Ito formula [25], we get

(B1)

By the fact that

(B2)

and , we get the following inequality after some rearrangements

(B3)

Then, following the same procedure as the proof of Proposition 1 in Appendix A, we can get the results of Proposition 2. i.e. we can get the same result as Proposition 1 except three extra terms due to the need to tolerate functional perturbations in (B3).

## Appendix C: proof of proposition 3

Now, we will derive the sufficient condition to ensure that the linearized synthetic gene network in (27) can attenuate the external disturbance below a prescribed attenuation level *ρ *in (17) or (18). By choosing a positive Lyapunov function as , we have

(C4)

By Ito's formula and , we have

(B5)

By the inequality in (28), we have

(B6)

Then, the remainder of the proof is similar to the procedure in Appendix A

## Appendix D

The global linearization technique can be employed to transform the nonlinear stochastic gene network into an interpolation of a set of globally linearized gene networks. In this design example, the global linearizations are bound by a polytope consisting of 3 vertices, shown as follows



where


















